# Supplementary material for: Coverage-guided differential testing of TLS implementations based on syntax mutation
Source: PLoS One. 2022 Jan 24;17(1):e0262176. doi: 10.1371/journal.pone.0262176 (PMC8786154; doi:10.1371/journal.pone.0262176)
Supplement: Attachment — Submitted filename: Response to Reviewers.docx [file pone.0262176.s003.docx]

Dear Editors and Reviewers:

On behalf of my co-authors, we appreciate editors and reviewers for their constructive comments and suggestions on our manuscript entitled “Coverage-guided differential testing of TLS implementations based on syntax mutation” (PONE-D-21-31321).

We studied reviewer’s comments carefully and made revision which marked in red in the paper. We tried our best to revise our manuscript according to the comments. The main corrections in the paper and the responds to the reviewer's comments are as flowing.

Responds to the reviewers’ comments:

Reviewer #1:

1. Response to comment: there are some editorial errors, such as P2 99.

Response: We are very sorry for our incorrect writing. We modified the sentence. Meanwhile, similar errors in the article were reviewed and corrected in detail.

2. In addition, figures should locate above the caption. It took me a lot of effort to find them.

Response: As the journal requires that figures are not included in the manuscript, we are very sorry for bringing you the inconveniences. We added the figure legend for every figure.

Reviewer #2:

1. In the article, there are many abbreviations which make reading difficult, so I suggest the authors give an explanation when they first appear, or list a table to explain them.

Response: For ease of understanding, we deleted non-standard abbreviations unless they appear at least three times in the text. Meanwhile, if the abbreviation is not well known, the full name of the abbreviation was given when it first appeared. In addition, we listed a table to explain the meanings of mathematic symbols often used in the paper.

2. The presentation quality is to be polished. Or I suggest the author to find a professional organization to polish English.

Response: Considering the Reviewer's suggestion, we found the professional organization “EditSprings” to polish English. We tried our best to improve the manuscript and made some changes in the manuscript. These changes will not influence the content and framework of the paper. And here we did not list the changes but marked in red in revised paper.

Once again, we appreciate for your comments and suggestions.

Thank you and best regards.

Sincerely yours,

Corresponding author: Yuefei Zhu

E-mail: yfzhu17@sina.com
